# A170 DISTRIBUTION OF PHARMACOGENETIC VARIANTS IN PEOPLE SUFFERING FROM ULCERATIVE COLITIS TREATED WITH TARGETED MOLECULAR THERAPIES IN QUEBEC

**DOI:** 10.1093/jcag/gwae059.170

**Published:** 2025-02-10

**Authors:** L Tessier, E Fortin, A Michaud-Herbst, K Tremblay

**Affiliations:** Universite de Sherbrooke, Saguenay, QC, Canada; Universite de Sherbrooke, Saguenay, QC, Canada; CIUSSS du Saguenay-Lac-St-Jean, Saguenay, QC, Canada; Universite de Sherbrooke, Saguenay, QC, Canada

## Abstract

**Background:**

Ulcerative colitis (UC) is a chronic inflammatory bowel disease characterized by continuous colonic inflammation. In Canada, ~160,000 people suffer from its symptoms, such as diarrhea, blood in the stool and abdominal pain, which can highly impact their quality of life. For moderate to severe forms of the disease, molecular targeted therapies (MTT) are used to control symptoms and achieve remission. There is a large variability in treatment response to MTT. Indeed, ~20-50% MTT users don’t improve following drug initiation and 50-90% suffer from adverse events. Multiple factors, such as age, sex, tobacco use and pharmacogenetic (PGx) variants, may influence response. However, genetic variants may also impact UC development which may become a confounding factor in treatment response studies.

**Aims:**

Therefore, the aim of this study is to validate PGx variants associated with nonresponse by comparing their distribution in UC patients with a populational cohort.

**Methods:**

A cohort of 101 people using MTT in UC in Saguenay–Lac-St-Jean was recruited and compared to the populational cohort of CARTaGENE (representing Quebec population, n = 1879). The patients were genotyped for eight PGx variants, distributed across four genes (*TNF*, *TNFAIP3*, *TNFRSF1A* et *TNFRSF1B*), which were selected as candidates for their previous association with MTT response phenotypes.

**Results:**

The variant *TNFRSF1B*-rs1061622, previously associated with nonresponse to infliximab (a type of MTT) in a previous study by our team, was comparable between the recruited and populational cohorts. The alternative allele of variant *TNFRSF1B*-rs3397 was more frequent in the recruited cohort compared to the populational cohort (80,0 vs 67,0 %, p = 0.0001). No other tested variants were differently distributed.

**Conclusions:**

Next steps are to compare these PGx variants with a populational cohort of healthy individuals (exclude individuals suffering from UC in CARTaGENE cohort) to eliminate as many confounding factors as possible. The variant *TNFRSF1B*-rs1061622 would then be validated and become a great lead to better comprehend treatment response to infliximab in UC. The variant *TNFRSF1B*-rs3397 needs to be studied more to clarify its potential link to UC development.

**Funding Agencies:**

CCCFRQS, IRSC, Fondation de ma vie

